# Assessing Differences in mHealth Usability and App Experiences Among Young African American Women: Secondary Analysis of a Randomized Controlled Trial

**DOI:** 10.2196/51518

**Published:** 2024-04-16

**Authors:** Claudia A Opper, Felicia A Browne, Brittni N Howard, William A Zule, Wendee M Wechsberg

**Affiliations:** 1 RTI International Research Triangle Park, NC United States; 2 Gillings School of Global Public Health University of North Carolina Chapel Hill, NC United States; 3 Department of Psychiatry and Behavioral Sciences Duke University School of Medicine Durham, NC United States

**Keywords:** HIV, Black women, mobile apps, social determinants of health, prevention, substance use, usability

## Abstract

**Background:**

In North Carolina, HIV continues to disproportionately affect young African American women. Although mobile health (mHealth) technology appears to be a tool capable of making public health information more accessible for key populations, previous technology use and social determinants may impact users’ mHealth experiences.

**Objective:**

The objective of this study was to evaluate mHealth usability, assessing differences based on previous technology use and social determinants among a sample of African American women in emerging adulthood.

**Methods:**

As part of a National Institute on Drug Abuse–funded randomized controlled trial with African American women (aged 18-25 years), counties were assigned to receive an evidence-based HIV risk reduction intervention through mHealth and participants were asked to complete usability surveys at 6- and 12-month follow-ups. Participants’ first survey responses were analyzed through 2-tailed *t* tests and linear regression models to examine associations with previous technology use and social determinants (*P*<.05).

**Results:**

The mean System Usability Scale (SUS) score was 69.2 (SD 17.9; n=159), which was higher than the threshold of acceptability (68.0). Participants who had previously used a tablet indicated higher usability compared to participants without previous use (mean 72.9, SD 18.1 vs mean 57.6, SD 11.4; *P*<.001), and participants with previous smartphone use also reported higher usability compared to participants without previous use (mean 71.9, SD 18.3 vs mean 58.0, SD 10.7; *P*<.001). Differences in SUS scores were observed among those reporting homelessness (mean 58.3, SD 19.0 vs mean 70.8, SD 17.2; *P*=.01), unemployment (mean 65.9, SD 17.2 vs mean 71.6, SD 18.1; *P*=.04), or current school enrollment (mean 73.2, SD 18.5 vs mean 65.4, SD 16.5; *P*=.006). Statistically significant associations were not observed for food insecurity (mean 67.3, SD 18.6 vs mean 69.9, SD 17.7; *P*=.45).

**Conclusions:**

Although above-average usability was observed overall, these findings demonstrate differences in mHealth usability based on past and current life experiences. As mHealth interventions become more prevalent, these findings may have important implications for ensuring that mHealth apps improve the reach of evidence-based interventions.

**Trial Registration:**

ClinicalTrials.gov NCT02965014; https://clinicaltrials.gov/study/NCT02965014

**International Registered Report Identifier (IRRID):**

RR2-10.1186/s12889-018-5796-8

## Introduction

In 1981, the first report was published identifying the disease that was later known as AIDS, marking the official beginning of the HIV epidemic [1]. In that same year, IBM’s first PC was sold to the public [2]. As the HIV epidemic persists, there may be an opportunity to embrace the digital age we are living in and leverage technological solutions as we work toward the shared goal of ending the HIV epidemic.

HIV incidence rates remain disproportionate based on race in the United States, as rates for Black or African American women are 10.9 times higher than rates for White women [3]. Further, the highest rates of HIV diagnoses occur in the US South [3]. Many interventions have been developed for Black or African American women [4-8], but barriers such as lack of transportation, limited childcare access, concerns over privacy, and community-level stigma impede access to HIV testing and prevention services [9,10].

Most Americans have smart mobile devices [11]. These devices show promise in diminishing barriers and connecting key populations with public health information through increasingly convenient and private pathways. Research has demonstrated that being a woman, young, and African American are characteristics associated with being more likely to prefer mobile health (mHealth) when given a choice or to use mobile devices to seek health information [12,13], supporting mHealth interventions as potentially effective tools for this key population.

However, individuals may experience and engage with mHealth interventions differently. As the prevalence of mHealth apps continues to increase [14], there is a need to understand mHealth usability. In a scoping review of electronic health applications from 2014-2017, the rate of new health applications available outpaced the rate of published usability studies [15]. The authors explained that while most digital health apps are developed commercially, the results of commercial usability studies are not typically published [15]. Because of the limited reported data for mHealth usability, this study examined the usability of an mHealth HIV prevention intervention among young African American women in the US South.

A previous study adapted a best-evidence, women-focused HIV behavioral intervention for young African American women (the Young Women’s CoOp), which demonstrated efficacy in reducing sexual risk [16]. In preparation for a trial to test intervention delivery, an mHealth version of the Young Women’s Coop was developed [17]. This analysis of usability scores for the Young Women’s CoOp mHealth app examines whether previous technology experience and social determinants are associated with mHealth experiences.

## Methods

### Overview

Analyses in this paper encompass an assessment of the usability of the Young Women’s CoOp intervention that was adapted to an mHealth platform. The parent study reached 652 young African American women (aged 18-25 years) in North Carolina who reported recent condomless sex with a male partner and substance use. A complete description of the parent study’s eligibility criteria and procedures can be found in the study protocol paper [18].

In a 3-arm randomized trial implementing a cross-over design, 3 counties were assigned to receive the in-person delivery of the Young Women’s CoOp intervention, the mHealth delivery of the Young Women’s CoOp intervention, or standard HIV counseling and testing. Among the enrolled sample, 197 women were in the counties assigned to receive the mHealth delivery, which consisted of a 1-on-1 mHealth orientation and an Android tablet preloaded with the app that contained the 2-session intervention. Following the orientation, a tablet was provided to each participant to take with them. The study team requested that tablets be returned at the 6-month follow-up appointment.

The usability of the mHealth intervention was evaluated using a modified version of the 10-item System Usability Scale (SUS) [19]. SUS scores range from 0 to 100, with scores above 68 considered above average [20]. To account for participants missing follow-up appointments, mHealth participants completed the usability survey as part of an audio-computer–assisted self-interview (ACASI) at both 6-month and 12-month follow-ups. For participants who completed the usability survey at both follow-ups, only their first (6-month) survey response was considered. ACASI was administered in person in an attempt to engage and collect responses from mHealth users who may have had difficulty using the tablet or lost the tablet and who may have had challenges completing a tablet-hosted survey.

Social determinants (homelessness, unemployment, food insecurity, and school enrollment) were measured at study enrollment. Social determinant variables were either assessed as dichotomous questions (homelessness and school enrollment) or recoded into dichotomous variables (unemployment and food insecurity). Homelessness, unemployment, and school enrollment assessed an individual’s current state and food insecurity asked about one’s household. Descriptive statistics and 2-tailed *t* tests were used to assess bivariate associations between social determinants and usability scores. Linear regression was conducted to examine these associations while controlling for previous tablet use and previous smartphone use. Analyses were conducted in Stata 17 (StataCorp) using the threshold of *P*<.05 for statistical significance.

### Ethical Considerations

The full study received approval from the Office of Research Protection’s Institutional Review Board at RTI International (IRB ID number: 13836). Further, committees from Wake County Human Services and Durham County Department of Public Health, along with administration from the Guilford County Department of Public Health, granted study approval. All participants provided written informed consent. Several procedures were instituted to protect the privacy and confidentiality of study participants, including all staff members involved in data collection and analysis signing and abiding by Staff Agreements of Confidentiality. Additionally, each participant was assigned a unique alphanumeric participant identifier to limit study data being connected to identifying information, such as name and contact information. Study participants were compensated for completing the baseline appointment with US $50 in gift cards, the 6-month follow-up appointment with US $70 in gift cards, and the 12-month follow-up appointment with US $100 in gift cards.

## Results

The overall mean SUS score was 69.2 (SD 17.9; n=159). Less than 12% (n=19) of participants did not have experience with a tablet or smartphone before the study. Variability of SUS scores by previous technology use and social determinants is shown in Table 1.

Participants who had previous tablet use reported higher SUS scores on average than participants who had not previously used a tablet (72.9, SD 18.1 vs 57.6, SD 11.4; *P*<.001). Similarly, participants who had previously used a smartphone had a higher mean SUS score than participants who had not (71.9, SD 18.3 vs 58.0, SD 10.7; *P*<.001).

Additionally, the mean SUS scores were under the acceptable threshold for participants reporting food insecurity, homelessness, unemployment, or no current school enrollment. Statistically significant differences in mean SUS scores were observed among those reporting homelessness (58.3, SD 19.0 vs 70.8, SD 17.2; *P*=.01), unemployment (65.9, SD 17.2 vs 71.6, SD 18.1; *P*=.04), or current school enrollment (73.2, SD 18.5 vs 65.4, SD 16.5; *P*=.006). Statistically significant associations were not observed in the SUS score based on food insecurity (67.3, SD 18.6 vs 69.9, SD 17.7; *P*=.45). When accounting for previous mobile technology experience in each model, homelessness and current school enrollment were statistically significant, but unemployment and food insecurity were not statistically significant (Table 2).

**Table 1:** Bivariate associations between System Usability Scale (SUS) score and previous technology use and social determinants of health.

		Frequency, n (%)	SUS score, mean (SD)	*P* value
**Previous tablet use**				<.001
	Yes	121 (76.1)	72.9 (18.1)	
	No	38 (23.9)	57.6 (11.4)	
**Previous smartphone use**				<.001
	Yes	128 (80.5)	71.9 (18.3)	
	No	31 (19.5)	58.0 (10.7)	
**Homelessness**				.01
	Yes	20 (12.6)	58.3 (19.0)	
	No	139 (87.4)	70.8 (17.2)	
**Unemployment**				.04
	Yes	67 (42.1)	65.9 (17.2)	
	No	92 (57.9)	71.6 (18.1)	
**Food insecurity**				.45
	Yes	41 (25.8)	67.3 (18.6)	
	No	118 (74.2)	69.9 (17.7)	
**In school**				.006
	Yes	77 (48.4)	73.2 (18.5)	
	No	82 (51.6)	65.4 (16.5)	

**Table 2:** Associations between System Usability Scale (SUS) score and social determinants of health, adjusting for previous tablet use and smartphone use.

Independent variables	Coefficient (95% CI)	*P* value
Homelessness	–9.0 (–16.8 to –1.1)	.03
Unemployment	–4.9 (–10.1 to 0.3)	.07
Food insecurity	–1.5 (–7.5 to 4.4)	.61
In school	6.2 (1.1 to 11.3)	.02

## Discussion

As mHealth continues to become more prevalent, these findings show an overall above-average usability for the mHealth adaptation of the Young Women’s CoOp intervention. Notably, there were differences in SUS scores based on a participant’s past experiences with technology. Although less than 12% of the study sample had not previously used a smartphone or tablet, this proportion was higher than what may be expected for this age group based on national survey data for young adults [11]. This finding suggests the importance of considering ways the digital divide and other factors may impact familiarity with mobile technology when designing mHealth interventions for this key population. In this study, before participants were given tablets with the mHealth intervention, they received a brief orientation to the app. Given the lower reported usability among those who lacked experience with mobile technology, these findings suggest examining further whether providing more guidance during app orientation could improve app usability for those with limited previous experience. Additionally, ongoing and other technology support (eg, a chat feature where trained staff can provide technology support to users within the app) may be strategies to explore with guidance from the intended end users to see if they improve mHealth experiences for individuals with less mobile technology experience.

Given privacy and stigma-related barriers that may hinder access to in-person HIV prevention programs and services for young African American women [9,10], mHealth could be an attractive solution. However, our findings exemplify that not only previous experience with technology but also diversity in participants’ life circumstances, such as homelessness and school enrollment, can be associated with usability. Though the format may appear well-positioned for young adults, it is imperative to consider how a surge in the use of mHealth may miss the opportunity to maximally address existing health disparities if some users encounter barriers when operating the mHealth app that undermine their experiences. Additional guidance and support will be essential for those with factors associated with lower usability.

This study should be considered in relation to a few limitations. All participants completed the intervention using a study-issued Android tablet. Noting how a device’s model or operating system may affect usage, some user experiences may have been shaped by the device specifications. In future usability studies, it may be valuable to have participants use their own devices to minimize the chance that device unfamiliarity affects assessments of app usability. Further, participants were asked to assess usability at 6- and 12-month follow-ups. Though participants still had access to the app before returning the device at their follow-up appointment, there was potential for recall bias as usability may have been assessed months after a participant’s last app interaction. Additionally, it should be noted that all experiences with the app and data collection occurred before the COVID-19 pandemic. With a greater shift to digital formats for health, education, social, and other services throughout the pandemic, access to mobile devices and familiarity with receiving information through mobile technology may have increased since this study.

Despite these limitations, the study prompts important considerations as the health sector embraces digital technology. The overall above-average usability score signals the potential value of using mHealth as a delivery method in the public health toolkit to further expand the reach of evidence-based interventions to those who may need it the most.

## Abbreviations

ACASI: audio-computer–assisted self-interview
mHealth: mobile health
SUS: System Usability Scale
